# Genomic and phenotypic analysis of Vavilov’s historic landraces reveals the impact of environment and genomic islands of agronomic traits

**DOI:** 10.1038/s41598-017-05087-5

**Published:** 2017-07-06

**Authors:** Elena Plekhanova, Margarita A. Vishnyakova, Sergey Bulyntsev, Peter L. Chang, Noelia Carrasquilla-Garcia, Kassaye Negash, Eric von Wettberg, Nina Noujdina, Douglas R. Cook, Maria G. Samsonova, Sergey V. Nuzhdin

**Affiliations:** 1 Department of Applied Mathematics, Peter the Great St.Petersburg Polytechnic University, St. Petersburg, Russia; 2 Federal Research Centre All-Russian N.I. Vavilov Institute of Plant Genetic Resources (VIR), St. Petersburg, Russia; 3 Department of Plant Pathology, University of California, Davis, CA USA; 4 Program Molecular and Computation Biology, Dornsife College of Letters, Arts, and Sciences, University of Southern California, Los Angeles, CA USA; 5 Department of Biological Sciences and International Center for Tropical Botany, Florida International University, Miami, FL USA; 6 School of Architecture, University of Southern California, Los Angeles, CA USA

## Abstract

The Vavilov Institute of Plant Genetic Resources (VIR), in St. Petersburg, Russia, houses a unique genebank, with historical collections of landraces. When they were collected, the geographical distribution and genetic diversity of most crops closely reflected their historical patterns of cultivation established over the preceding millennia. We employed a combination of genomics, computational biology and phenotyping to characterize VIR’s 147 chickpea accessions from Turkey and Ethiopia, representing chickpea’s center of origin and a major location of secondary diversity. Genotyping by sequencing identified 14,059 segregating polymorphisms and genome-wide association studies revealed 28 GWAS hits in potential candidate genes likely to affect traits of agricultural importance. The proportion of polymorphisms shared among accessions is a strong predictor of phenotypic resemblance, and of environmental similarity between historical sampling sites. We found that 20 out of 28 polymorphisms, associated with multiple traits, including days to maturity, plant phenology, and yield-related traits such as pod number, localized to chromosome 4. We hypothesize that selection and introgression via inadvertent hybridization between more and less advanced morphotypes might have resulted in agricultural improvement genes being aggregated to genomic ‘agro islands’, and in genotype-to-phenotype relationships resembling widespread pleiotropy.

## Introduction

A defining challenge of the 21st century is meeting the nutritional demands of a growing human population, using increasingly limited land and water resources and under the spectre of climate change^1^. Agriculture must simultaneously intensify, become more sustainable, and achieve greater resilience to pests and climate. New paradigms are needed to increase sustainability in agricultural systems, including methods to explore the genetic potential of the vast but woefully underutilized germplasm resources available for most crop species. Crucial to this effort are the recent advent of low cost, high throughput DNA sequencing technologies and corresponding advances in computational genomics^2^.

However, the above tools are only helpful when they are applied to appropriate germplasm. The Vavilov Institute of Plant Genetic Resources (VIR) in St. Petersburg is a uniquely valuable collection of crop germplasm because it captures the genetic and functional diversity of regionally stratified agriculture typical of one century ago. The VIR’s focus on locations of historical chickpea domestication in the Middle East and of long-standing secondary centres of diversity, including in Ethiopia^3^ provide access to millennia of human-selected adaptations present in landraces. The majority of such genetic diversity has been removed from modern agricultural systems through Green Revolution practices^4^. Here we combine genomics, computation and phenotyping to characterize molecular and phenotypic variation in a sample of the collection of chickpea (*Cicer arietinum* L.) amassed by Nikolay Vavilov and his colleagues, linking valuable adaptations to genome intervals and candidate genes and resurrecting the collection’s currently latent power to meet the enormous challenges of 21st century agriculture.

At the beginning of the 20th century, leading agronomists and geneticists recognized the need to preserve and characterize the genetic diversity of cultivated plants and their wild relatives. D. Fairchild and his staff in the USA and many others organized expeditions to sample biodiversity, but it was Nikolay Vavilov and the considerable resources committed by the growth-hungry post-revolution Soviet government that made the largest contribution^5, 6^. Vavilov contributed importantly to the paradigm that domestication of crops occurred at the species’ ‘centre of origin’, where recurrent selection of the most valuable plants, from generation to generation, resulted in genetic divergence and isolation from wild progenitors, ultimately yielding domesticated species. As cultivation spread regionally, hundreds to thousands of locally distinct forms arose through further selection, drift and gene flow, generating landraces. Landraces dominated agriculture from ~7 KYA until the advent of intensive modern breeding in the mid 20th century – when a few elite cultivated varieties largely displaced landraces.

Grain legumes, including chickpea, are the primary source of nutritional nitrogen for approximately 30% of the world’s human population, and their consumption contributes to healthy lifestyles^7^. However, legumes were not equal beneficiaries of the Green Revolution. Policy and investment since the 1960’s favoured Green Revolution cereal crops, which were planted on the best agricultural land and received the lion’s share of inputs. Legumes, on the other hand, were often relegated to marginal lands where elevated temperatures, rainfed cropping systems, short growing seasons and poor soils conspire to limit yield potential^8^. Current grain legume production (e.g., chickpea, common bean, groundnut, lentil, and pigeonpea) in impoverished, food-insecure countries is often significantly short of demand. Simultaneously, modern breeding has collapsed the historical diversity of crops like chickpea. The massive reduction in genetic variation^9^ constrains crop improvement and genetic gain.

The VIR collection reflects local crop diversity before the intensification and global homogenization of modern breeding efforts, which have tended to focus on a narrower and narrower set of improved lines^5, 6, 10^. Knowledge of the genomic basis of phenotypic variation in relatively diverse landrace collections, such as those of the VIR^11^, will further enable crop scientists to devise solutions to agricultural constraints. The VIR houses 928 accessions of chickpea sampled by Vavilov and his colleagues between 1911–1940, with corresponding phenotypic data collected at semi-regular (2–5 year) intervals during recent decades. Our goal was to test the feasibility of combining genomics and computation with historical records to mine the VIR collection for “genomic gems” that might offer solutions to the tremendous challenges of modern agriculture, including increased productivity with decreased environmental impact. Towards this aim, we present analysis of a subset of 147 landrace accessions for which complete phenotypic data replicated over time are available (see Methods).

## Results

To start analysing the wealth of the VIR germplasm coupled with available phenotypic data, we have limited our scope to the oldest chickpea accessions collected nearly a hundred years ago from one centre of primary chickpea domestication (Turkey) and one centre of secondary diversification (Ethiopia)^11^. We have obtained reduced representation sequencing data for these 147 landraces and combined these genomic data with ecogeographic and phenotypic data to deduce patterns of genomic variation and their association with trait values and the sampling environmental data. We first characterize genetic variation among these accessions, then show phenotypic variation, and finally connect genetic, phenotypic, and environmental data via the analysis of covariance between genetic and phenotypic resemblance, and via GWAS.

### Genotyping and Population Analysis

**Figure 1:**
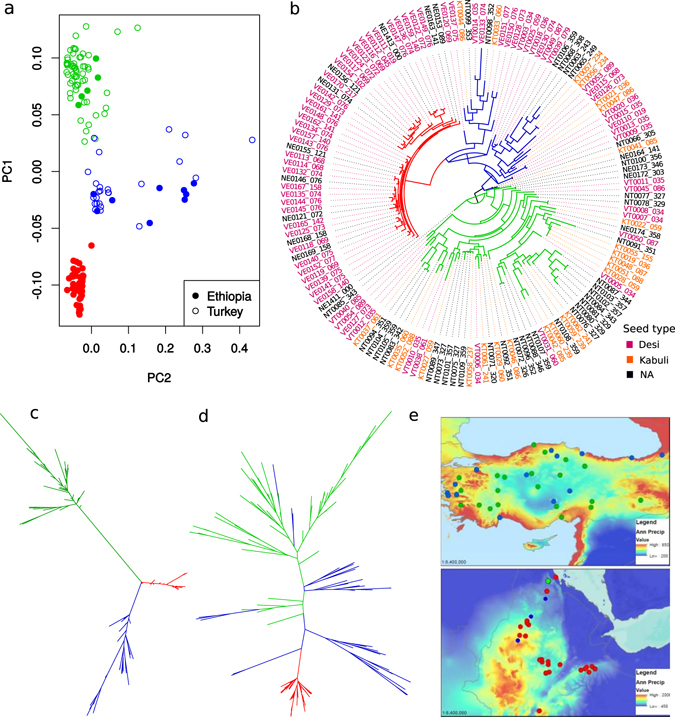
Analysis of 14,059 genome-wide SNP reveals patterns associated with chromosome of origin, geographical distribution, and a secondary bottleneck from Turkey to Ethiopia. Colour scheme in all panels of the figure (red, green and blue) corresponds to separation of accessions on PC-plot (**a**), into three clades on whole genome tree (**b**) and into three clades on chromosome 4 tree (**c**). (a) Principal component plot constructed for all SNPs separates accessions by the country of origin (Ethiopia and Turkey) and reveals three clusters that correspond to genetic structure revealed on maximum likelihood tree of all accessions shown in (**b**). (**b**) Maximum likelihood phylogenetic tree for the chickpea landraces based on the whole genetic material. (**c**) Maximum likelihood phylogenetic tree showing relationships among accessions based on chromosome 4 SNPs, and (**d**) the rest of the genome. (**e**) Geography of accessions with origin in Turkey or Ethiopia^84^, (the map was created with ArcGIS 10.3.1 software, http://www.esri.com/). Map coloration depicts annual precipitation – a variable predictive of the plant phenology. Twenty of 45 Ethiopian accessions were collected in Addis Ababa and appear as a single point on the graph.

Genotyping-by-sequencing identified 14,059 segregating SNPs among 147 accessions originating from Turkey and Ethiopia. Principal component analysis (PCA) revealed two eigenvectors separating landraces by country of origin (see Fig. 1a). On the other hand, we observe three clusters (Fig. 1a) that correspond to genetic structure exhibited by the maximum likelihood tree (Fig. 1b). To test whether all chromosomal regions show these patterns, we repeated these analyses by chromosome. We observed that SNPs from the chromosome 4 (Fig. 1c) delimit the above three well-resolved groups, whereas the remaining genome-wide SNPs produced less clearly resolved patterns (Fig. 1d).

The patterns above originate from a combination of geographic subdivision, genetic bottlenecks and/or interbreeding, and also potentially reflect different types of biotic or abiotic selection. The history of chickpea domestication and breeding involved differentiation of the crop into two market classes based on a suite of traits, typified by differences in seed size, seed coat tannins and flower colour^12–16^. Desi genotypes generally have smaller, dark seeds and coloured flowers, while Kabuli genotypes are characterized by larger, light coloured seed and white flowers. Based on the analysis of molecular markers, patterns of geographic distribution, and resemblance to the wild progenitor species, Desi is accepted as the ancestral state^12–15^. With this knowledge, and observing patterns shown in Fig. 1, we propose two observations. The subdivision between green and blue genotypes in Fig. 1 is basal, arising at the centre of primary domestication in Turkey, not correlated with Desi-Kabuli differences (Fig. 1b), and most pronounced on the 4^th^ chromosome. Only the blue clade migrated to Ethiopia (with a single exception, potentially due to mislabelling), where it gave birth to the low diversity red cluster apparently involving a secondary genetic bottleneck.

To consider whether these observations are consistent with the patterns of molecular evolution, we rooted the tree using the wild progenitor, *C*. *reticulatum*, as an outgroup (Fig. 1b). The resulting tree topology underscores the origin of Ethiopian genotypes as derived from the blue Turkish clade, while the dispersion of Kabuli forms among the green and blue Turkish clades supports our earlier conclusion that the Kabuli form is polyphyletic^16^.

### Phenotypic Analyses

While VIR germplasm accessions were regrown at consistent intervals of 2–5 years, the corresponding phenotypic records are sometimes shallow and fragmented. Luckily, these accessions of chickpea have been a subject of two detailed analyses, as described in more detail by Vishnyakova *et al*.^11^. These phenotyping experiments were performed at Aleppo, Syria (69 accessions) and Astrakhan, Russia (109 accessions). We felt that an attempt to analyse this data would be highly informative as a ‘proof of concept’, illustrating whether useful inferences might be derived from the VIR historic data. Unfortunately, the lists of phenotypes recorded were overlapping but not completely consistent between these phenotyping experiments. Accordingly, we characterized phenotypic variation separately at each phenotyping site using factor analysis (FA).

**Figure 2:**
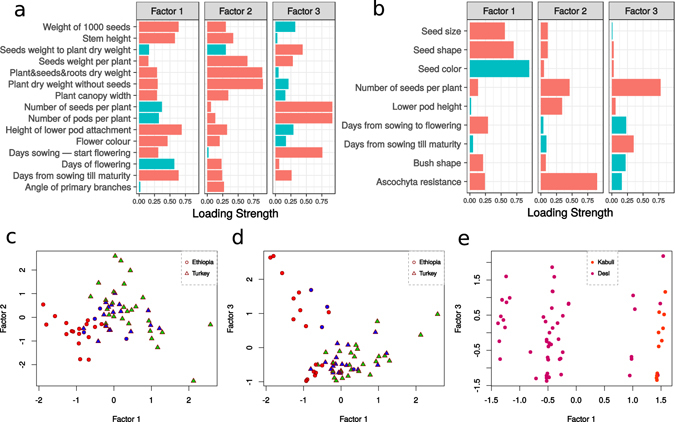
Factor analysis of phenotypic data reveal correlated traits. Factor loadings for Syrian field trial (**a**) and Astrakhan (Russian) field trial (**b**) phenotype data (red colour corresponds to positive loadings while turquoise colour to negative ones). (**c**) and (**d**): Factor 1 of Syrian phenotype data separates accessions according to colour scheme in all panels of Fig. 1. Green and red clusters are phenotypically distinct, while blue and green clusters partially overlap. As Factor 1 is driven by seed size and flower colour characteristics that differ between Desi and Kabuli varieties, it distinguishes market classes. (**e**) Factor 1 of Astrakhan phenotype data separates accessions largely based on seed characteristics that distinguish Kabuli and Desi varieties.

FA on the Syrian data identified 3 axes that account for 57% of phenotypic variation (Fig. 2a). Factor 1 associates longer vegetative growth with taller plants and larger seeds, as well as flower colour (Fig. 2a). This factor separates accessions according to country of origin and population clusters (Fig. 2c,d). Factor 2 is driven largely by plant and seed biomass, demonstrating strong covariance between non-reproductive and reproductive organs. Factor 3 has primary loading from plant reproduction, i.e., days to flowering, number of pods and seed per plant.

Three factors also account for a significant proportion (49%) of the observed variation for the Russian field data (Fig. 2b). Seed characteristics were the primary properties associated with Factor 1, while resistance to *Ascochyta* blight, a devastating foliar disease of chickpea, was the primary phenotype loading onto Factor 2. As was the case for the Syrian analyses, Factor 3 was most associated with plant fecundity (Fig. 2b). Interestingly, Factor 1 from the Russian data set clearly differentiates Desi from Kabuli forms, consistent with major loading from seed size, shape and colour characteristics (Fig. 2e).

### Genotype-to-phenotype map

The degree to which genotype differences explain phenotypic variation depends both on the density of segregating sites and the extent of linkage disequilibrium (LD). If LD extends beyond several GBS loci, then most genome-wide associations should be captured. Our GBS protocol samples ~3% of the genome, which after QC and filtering of mapped reads yielded 14,059 SNPs with known genome locations. Does it yield a map dense enough to analyse genotype-phentoype associations?

**Figure 3:**
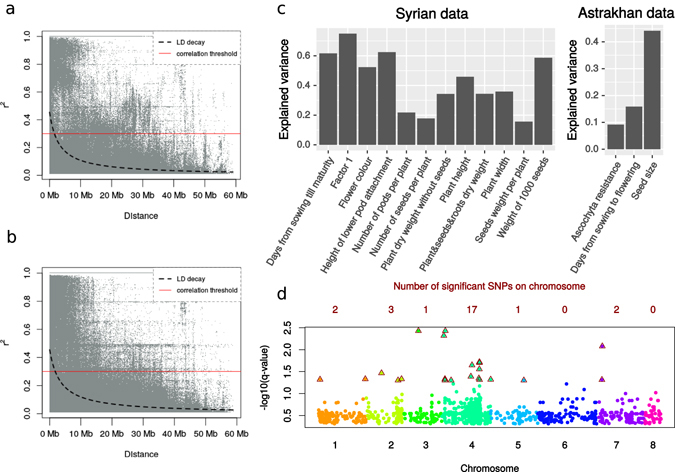
Genomic analyses reveal genetic control of traits and an enrichment of trait associations on chromosome 4 (**a**) LD measured by r^2^ as a function of genetic distance between SNPs for all landraces and (**b**) for landraces from Turkey. (**c**) Proportion of phenotypic variance explained by genotype for different traits. y-axis ratio of genetic variance to phenotypic variance of a trait, x-axis – different trait phenotypes. (**d**) Summary of GWAS analyses for Astrakhan (Russia) and Allepo (Syria) phenotype data (different colours corresponds to different chromosomes). SNPs with q-value < 0.05 are shown for each chromosome, marked as triangles. When one position associates with a number of phenotypes with different q-values, only the most significant SNP is represented.

The analysis of LD decay with the distance between SNPs establishes that significant LD remains up to approximately 1.5 Mb (Fig. 3a), a pattern consistent with population admixture and recombination (see Fig. 3b for Turkish accessions and Supplementary Figure S1 for analysis for Ethiopian accessions). Overall, we conclude that the distance between GBS markers is much shorter than a typical LD block, thereby providing sufficient coverage to identify genotype-to-phenotype associations.

Here we first tested whether genetic resemblance, as measured by the proportion of sites shared between accessions, predicts phenotypic resemblance among the accessions. Note, this is akin to ‘genomic heritability’^17, 18^, however our inferences must be treated with substantial caution due to a limited sample size. Both Syrian and Astrakhan phenotyping data reveal strong association between genomic and phenotypic resemblance for most phenotypes (those significant at p-value < 0.05 are shown in Fig. 3c; see also Supplementary Table S2).

We next moved to search for strong-effect QTLs controlling plant phentoypes. While, once again, our sample size is small, plant QTLs frequently have strong effects, and they are routinely detected with sample sizes in low hundreds of individuals^19^. Figure 3d provides a summary of GWAS analyses (also see Supplementary Figures S2 and S3, and Supplementary Table S3), exhibiting the detection of 28 GWAS hits, 20 of them on the 4^th^ chromosome. One explanation for this overabundance could be higher probability of QTL detection due to denser SNPs. We tested this hypothesis by comparing chromosomes, and did observe nearly two fold comparative excess of the SNPs on this chromosome (Supplementary Figures S4 and S5). As even in the least marker-dense regions of the genome, LD typically exceeds the distance between markers, we reject this conjecture.

For the Syrian data the GWAS analysis identified 13 SNPs significantly associated with different phenotypes. Nine of these SNP localize to chromosome 4, among which seven SNPs (see Supplementary Tables S3 and S4 for details) associate with multiple traits and localize to a region of 620 Kb with strong linkage. All seven SNPs are strongly associated with days from sowing to flowering (Days sowing – start flowering) and branches angle phenotypes, some associate with plant canopy width, flower colour phenotypes, seed weight to plant dry weight, stem height, height of the lowest pod attachment, and seed weight to plant dry weight. An additional set of significant associations was found by analysis of the Russian phenotypic data, for which we observed 13 SNPs strongly associated with seed characteristics, namely size, colour and shape, with eight of these SNPs localized to different regions on chromosome 4.

The approach of combining data from landraces, sampled at two centres of biodiversity (primary – Turkey, and secondary – Ethiopia) for which population subdivision is strong, is likely to inflate the apparent statistical significance of our inferences. To control for this possibility, we generated q-q plots and observed little evidence for such an inflation (see Supplementary Figures S2 and S3). Further, given that the first two principle components account at large for population effects (see Fig. 1a), we repeated the GWAS analyses including PC1 and PC2 into the model. Two SNPs, Ca2:15637875–15639356 (Ca_18541) and Ca3:16382686–16382988 (Ca_18260) associated with seed and pod number^20^, remained significantly associated. The initial associations reported above might be real as well, because the limited number of genotypes under analysis necessarily reduced power for detection, which can be rectified by increasing the number of accessions in the study.

### Candidate Genes

The associations we have uncovered mapped to broad genomic regions because of extended LD. They cannot identify causal relationships between SNPs and phenotypes. Nevertheless, it is of interest to explore potential nature of the associated genes. We report that among 28 significant SNPs, 12 are located in gene sequences and 12 near gene sequences with assignable protein functions. Two of these genes encode protein domains reported to contribute to DNA repair, recombination and/or replication; two have domains involved in plant growth and development; nine contain domains with roles in signal transduction (serine-threonine-tyrosine protein kinases, histone modifying proteins, transporters); and four encode proteins participating in plant defence responses, namely cell wall modification, cell death, redox reactions, immune response and detoxification (see Supplementary Table S4). For example, Ca4:37658225–37661428 (Ca_15114) contains three SNPs associated with multiple traits, and is 42.4% identical to the *Medicago truncatula* LYSM receptor-like kinase, LYK3, a gene with known roles in nodulation and response to pathogens^21^. Two SNPs are located in Ca4:37824762–37828199 (Ca_15093), annotated as an ATP-dependent DNA helicase RecG that plays a critical role in recombination and DNA repair in bacteria^22^. Plant DNA helicases are likely to have a variety of roles, but a phenotype they have been linked to of relevance here is plant growth and development^23^ and response to abiotic stress. The chloroplastic RecG of *Arabidopsis thaliana* (At2g01440) is 70.7% identical to the Ca_15093 protein and is differentially expressed in response to stress, including anoxia, cold, drought, genotoxic, hypoxia, heat, osmotic, oxidative, salt and wounding^24^. Ca4:30295389–30312868 (Ca_14192) encodes a putative chromatin remodelling protein from the sucrose non-fermenting 2 (Snf2) family of DNA helicases/ATPases and is 63% identical to *A*. *thaliana* PIE1 (Photoperiod-Independent Early flowering 1). PIE1 belongs to the Swr1 subfamily of Snf2 proteins and is believed to maintain negative control of the salicylic acid-dependent defence pathway^25–27^. Another gene, Ca1: 46968371–46976822 (Ca_12942), encodes trehalose 6-phosphate synthetase. Alteration of the amounts of trehalose 6-phosphate and/or trehalose can modulate abiotic stress tolerance. Each of these gene variants could plausibly underlie adaptation to environmental factors.

### Does ecogeography of landraces predict phenotypes?

**Figure 4:**
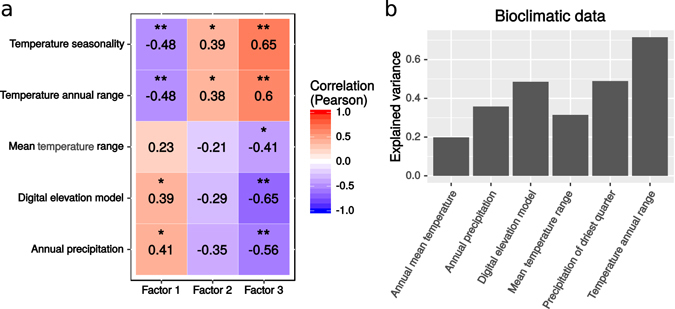
Phenotypic and genomic patterns correlate with bioclimatic variables. (**a**) Correlations between bioclimatic variables and Factors of Syrian phenotype data (*p-value < 0.05; **p-value < 0.01). (**b**) Genomic heritability of bioclimatic variables.

To determine if the variable phenologies of VIR landraces match environmental parameters in the sampling sites in Turkey and Ethiopia, we tested for covariance of phenological factors and bioclimatic variables at the accessions’ sampling sites (Fig. 1e). By way of example, we highlight the analysis of the Syrian phenotypic data (Fig. 4a), with a similar analysis presented for the Russian data in Supplementary Table S5.

For Factor 1, which is largely associated with plant height and seed/flower variation typical of the Desi - Kabuli split, we observe moderate significant negative correlation of flowering time with temperature, and positive correlation with annual precipitation and elevation (Fig. 4a). For Factor 2, which corresponds to biomass-related characters, we observe positive correlations of dry weight of plant and weight of seeds per plant with temperature. Positive correlations with temperature variation were also found for Factor 3, which is associated with plant reproductive phenotypes, while negative relationships were observed with elevation, mean diurnal range and annual precipitation. We conclude that plants with different phenologies occupy non-random subset of environments.

Given these tantalizing phenotype to environment associations, we further considered the environment at the sampling site as an extended phenotype of the accession. We hypothesized that germplasm sampled from environmentally similar sites would have high genome-wide relatedness. Indeed, genetic relatedness was a strong predictor of environmental similarity (Fig. 4b, see significant correlations in Fig. 4a). There might be two reasons behind such an observation. First, nearby plants might share common ancestry, with genotypes distributed locally for convenience and by custom (isolation by distance). Second, historical farming practices might have selected accessions with favourable phenological characters, thus distributing genetically similar plants among like environments (called G*E covariance). It appears, from inspecting Fig. 1d and e that accessions from different genomic clusters are geographically interspersed. However a more formal hypothesis testing must be implemented before accepting the hypothesis of G*E covariance over isolation by distance.

## Discussion

At the onset of Vavilov’s efforts near the turn 20^th^ century, landraces, with their idiosyncratic local properties, were the predominant type of cultivar in agriculture. Vavilov’s effort to sample extensively at crop centres of origin and at sites of secondary diversity was prescient. His goal was to archive the vanishing genetic diversity of crops for future generations of breeders. He wrote: “However rich nature might be in forms, the combinations of characters that would perfectly suit man would be extremely rare, and the deliberate creation of new and agriculturally more advanced forms constitutes a current objective of plant science. The recent experiments in genetics have unveiled much more opportunities than a researcher of the past could only dream about. In the near future man will be able to synthesize forms completely unimaginable nature”. Written in the 1920s but only published in 1951^28^, these ideas constitute a cornerstone of modern plant breeding^29^. The work described here on chickpea serves to demonstrate the power that genomics, phenotyping and computational analyses have to fulfil Vavilov’s vision. Collections such as those at the VIR, which is largely unique in historical breadth and depth, provide an unparalleled opportunity to understand the richness of agricultural diversity and function prior to the globalization of agriculture and science-based interbreeding and selection.

Understanding the genomic and environmental differences in crop domestication and post-domestication divergence, which likely differ by intensity and type of selection and the time frame under analysis, represents an important objective necessary to fully exploit landraces and wild species for sustainable food production in future agriculture. Early human selection on domesticated forms must have included strong selection for a handful of essential traits, such as non-shattering seeds, plant architecture, and perhaps phenology. Due to this striking phenotypic differentiation and the strong selection that likely accompanied it, traditionally the process of domestication was envisioned as starting from a genetic bottleneck that limited gene flow between wild and domesticated forms^30^. However, with growing evidence in some crops of prolonged gene flow between wild and cultivated forms^31^, some but not all recent thinking expand the traditional view by invoking the role of large, sympatric populations, with the possibility of prolonged gene flow both among domesticated subsets and from wild species^32, 33^. While gene flow homogenizes genetic divergence between wild and domesticated populations, selection reinforces such divergence – a plausible outcome is the concentration of domestication-driving genes in so-called ‘domestication islands’, akin to the ‘speciation islands’ of mosquito^34^. Implicit in this model is the concept that while several domestication alleles might arise, those controlling complex traits or that comprise independent but co-selected sets of traits are most likely to persist through gene flow and recombination if they are in linkage disequilibrium at one or a few genomic locations. Strong co-localization of QTLs affecting multiple traits in several regions of the pig genome lends impressive support to this view.

Here we describe a genotype-to-phenotype map of agricultural traits in chickpea using a subset of historic landraces preserved in the VIR collection that shows a strong pattern of localization of GWAS hits to one chromosome. Out of 28 GWAS hits, 20 mapped to chromosome 4. Nearly all these hits exhibited significant associations with multiple phenotypes, some of which are divergent between Desi and Kabuli market classes (Table 1). We note that Desi and Kabuli differ in multiple consumer-preferred traits, with Desi being the ancestral form and Kabuli a polyphyletic assemblage^16^ (Fig. 1b). These phenotypes segregate in patterns that are not random and are shaped along very few dimensions, suggestive of a co-selected complex resulting from co-inheritance of numerous genes.

**Table 1:** Differences between Desi and Kabuli seed types in allele frequencies of GWAS SNPs.

Chromosome	Position	Allele	Desi allele frequencies	Kabuli allele frequencies	P-value
1	1732351	C/G	0.09/0.91	0.46/0.54	2.368e-08
2	32154998	T/A	0.98/0.02	0.52/0.48	6.521e-15
4	2145082	T/G	0.98/0.02	0.38/0.62	6.286e-21
4	3235996	A/T	0.85/0.15	0.23/0.77	3.992e-15
4	3242507	G/A	0.90/0.10	0.24/0.76	1.414e-15
4	3302269	A/G	0.81/0.19	0.19/0.81	5.547e-10
4	9410036	T/C	0.81/0.19	0.27/0.73	3.088e-11
4	29186930	C/A	0.63/0.37	0.16/0.84	4.740e-06
4	30315118	G/C	0.81/0.19	0.31/0.69	7.545e-10
4	37188483	T/C	0.29/0.71	0.00/1.00	5.539e-05
4	37659499	G/A	0.42/0.58	0.05/0.95	5.447e-05
4	37659516	A/G	0.42/0.58	0.05/0.95	5.447e-05
4	37659524	G/A	0.44/0.56	0.05/0.95	1.762e-05
4	37805026	T/C	0.42/0.58	0.10/0.90	6.665e-05
4	37824651	C/G	0.43/0.57	0.10/0.90	8.924e-05
4	37824675	C/G	0.43/0.57	0.10/0.90	8.923e-05
4	37878401	A/G	0.43/0.57	0.09/0.91	0.0001
4	48998832	T/C	0.91/0.09	0.67/0.33	0.0001
5	33132457	T/A	0.71/0.29	0.96/0.04	0.001
7	5407505	G/T	0.42/0.58	0.95/0.05	1.257e-08
7	5782593	C/G	0.41/0.59	0.94/0.06	5.922e-10

Chi-square test showed that 21 of 28 significant SNPs have significant associations (p-value < 0.05) with either Desi or Kabuli market class. Fisher’s exact test was used for alleles with less than 10 representatives.

We posit that as increasingly advanced varieties accrue through selection on multiple genes, the cost of losing desirable trait complexes through outcrossing with a less advanced form also increases. Co-localization of co-adapted gene complexes in these more derived varieties theoretically mitigates such risk, enabling agricultural advance by reticulate processes, rather than simple fixation and linear descent. We nickname such gene complexes genomic ‘agro islands’. The existence of ‘agro islands’ is also strongly supported by prior data in chickpea. A comprehensive analysis of QTL region contributing to Ascochyta blight resistance that span 3 Mb of 2^nd^ chromosome led to identification of 306 genes, including genes typically involved in host resistance mechanism^35^. An analysis of traits associated with salinity tolerance revealed two key genomic regions on 5^th^ and 7^th^ chromosome, that harbor QTLs for yield in the salinity treatment. These regions span 11,1 Mb and 8.2 Mb on chickpea reference genome correspondingly and contain forty-eight (31 on chromosome 5 and 17 on chromosome 7) putative candidate genes known to play a direct or indirect role in osmoregulation that protects the plants not only from salinity stress but also from other abiotic stresses^36^. Kale *et al*.^37^ localized QTLs between two accessions – one Kabuli and another Desi – and mapped a variety of genes for drought resistance, mostly to the 4^th^ chromosome. Interestingly, the 4^th^ chromosome was found to have the maximum polymorphism (SNPs and SV) rate and maximum density of exonic variants when 35 chickpea genotypes representing parental lines of 16 mapping populations segregating for abiotic, biotic and nutritionally important (protein content) traits were re-sequenced^38^. Such studies lend support to our broader findings of numerous genes for multiple phenotypes mostly co-localized to a single genomic region. Interestingly, while the evolution of this genomic region has taken place in the geographic region of primary domestication, the evolved genotypes have not gained widespread representation in Ethiopia, which represents an important location of chickpea’s secondary diversification^39, 40^.

Is clustering of alleles contributing to local adaptation and domestication common or an exception? It was in fact demonstrated and discussed in numerous studies^34, 41–47^ (for review of genomic divergence in different species see Strasburg *et al*.)^48^. The proposed explanations included that these genomic islands of divergence could likely form through either genomic rearrangements that bring co-adapted loci close together or because the probability of a new mutation establishing is higher when occurring near another locally adapted mutation^49, 50^. However, most of the conclusions were derived through the genome scans, i.e. identification of outliers, that is subject to some statistical concerns.

In more detailed analyses, for example in populations of the violet species *Viola cazorlensis*, the divergence in floral traits that is potentially under pollinator-mediated selection was significantly associated with large number of loci^51^. There are also numerous small genomic regions underlying differentiation of sunflower species, and they are typically associated with low recombination rate^52^. One notable exception is association mapping of shoоt branching that plays an important role in sunflower adaptation to environment^53^. А large number of SNPs associated with branching map to single wide region of chromosome 10, where B locus responsible for branching is present as a large haplotypic block. This locus was reintroduced into sunflower gene pool to extend the flowering time in R lines. In maize, the loci implicated in domestication are spread around the genome; however there are also a few gene clusters^54, 55^ controlling a large portions of the phenotypic differences^56^. An idiosyncratic pattern of gene clustering was found on the fifth chromosome, it is associated with a number of domestication traits. Lemmon and Doebley^57^ demonstrated that this region may be split into multiple QTLs, none with singularly large effects. In rice domestication loci are also spread across the genome^58^, although the complexity of this domestication and the potential for ongoing gene flow with the wild relatives means fewer “islands” might have been involved initially. Indeed, many QTLs associated with rice domestication traits map to third chromosome. Significantly reduced nucleotide variation in genomic regions corresponding to these QTLs was demonstrated for one rice domesticated varietal type, *tropical japonica*. Coalescent simulations based on a complex demographic model inferred from genome-wide patterns of nucleotide variation suggested the third chromosome QTL regions might have been selected in this varietal type^59^. Interestingly, intron 1 splice donor site mutation in the *Waxy* gene that leads to the absence of amylose played a critical role in the origin of low amylose non-glutinous *temperate japonica* varieties. A large gene cluster that spans more than 250 kb and contains 39 genes including *Waxy* is due to selective sweep in this variety associated with the *Waxy* mutation^55^. Overall, more efforts will be needed to understand how common genomic islands are in plants. We require information from a greater number of domesticated crops, varying in center of origin, mating system, and agroecology to better understand the frequency with which genomic islands are involved in domestication.

Since we found that phenotypic variation is shaped into several ‘composite traits’ captured by our factor analyses, we studied whether such composite traits assort among environments. One might hypothesize that a certain value of a composite trait will fit one environment, while not matching another. For example, longer vegetative growth resulting in larger plants with higher yields might be maladaptive in localities with more pronounced seasonality or reduced rainfall. Farmers would likely avoid such maladaptive trait complexes, which would be evidenced as phenotype-environment co-variance. Indeed, such covariance appears frequently in our data set. We observed strong correlations of several environmental parameters with genomic resemblance. This hints that the preponderance of co-localized gene complexes might be co-adapted for a subset of cultivated environments. Whether this hypothesis holds for most domesticated crops (or even for the remainder of VIR’s extensive chickpea landrace collection) can now be tested using the hundreds of thousands accessions hosted at VIR, and for similar collections housed elsewhere, representing a remarkable opportunity to mine extant, but underutilized genomic gems. The resulting discoveries will contribute to meeting current and future agricultural challenges, including feeding a growing world, with nutritious outputs, in the face of increasing climatic variability and stress.

## Methods

### Germplasm

The 147 accessions under study were collected from two countries of origin. Eighty-four plants were from Turkey and 63 were from Ethiopia (see Phenotypes + environment table, page “genetic_information”; Supplementary Table S1), 80% of plants were collected from 1924 to 1928, others were collected after 1949. Note that 20 accessions are labelled as derived from Addis Ababa market, we retained all of them in the analyses as – most likely – they had originated from multiple nearby destinations but were taken to the Ethiopian capital for trade purposes^28, 29^. Reanalysis of the data with these accessions dropped results in general decrease of power but qualitatively similar conclusions.

### Phenotypic Data

Phenotypic data (see Phenotypes + environment table) were compiled for different traits, translated from VIR’s^11^ chickpea collection. The whole collection of 1082 accessions originated from 60 countries and thus representing global diversity of the crop. Here only the landraces from Turkish and Ethiopian landraces were considered. Of 147 accessions under study, only 109 were phenotyped (see Supplementary Table S1).

Two different subsets of traits were collected from two sites (Syrian and Astrakhan data), so we analysed these two sites separately. One subset of traits was assessed during the period 1996–2004 at VIR’s field experimental station in the Astrakhan region, Russia. At Astrakhan measurements of seed size, seed shape, seed colour, days from sowing to maturing, days from sowing to flowering, bush shape, height of lower pod attachment, Ascochyta tolerance, number of seeds per plant were performed. The second subset of traits of these accessions was assessed during the period 2000–2005 in the water-limited environment of Syria, at ICARDA’s Tel Hadya Research station near Aleppo. In Aleppo days of flowering, days from sawing to flowering beginning, yield index (the weight ratio of seeds to the dry weight of all plant, %), number of pods per plant, number of seeds per plant, dry weight of plant without seeds, seed weight per plant, dry weight of plant with seeds and roots, weight of 1000 seeds, days from sowing till maturity, plant canopy width, height of lower pod attachment, stem height, branches angle, flower colour were measured.

In both sites the accessions were planted in a randomized block experiment design, with two replications. Six plants of each replication were analysed. Soil cultivation and agricultural machinery matched the requirements of chickpea. In Astrakhan soils were heavy alluvial-meadow, loams. Sowing was carried out in late April and harvesting in late July to early August. With row spacing of 40 cm, distance between seeds was 6–7 cm. During the growing season six irrigations with sprinkling machines had been conducted and two mechanized processing of row spacing. At Tel Hdaya field station red soils were used. Plants were sown in February and harvested in August. With row spacing of 60–70 cm, distance between seeds was 10 cm. Soils were red soils. Artificial irrigation was absent. Field assessment was carried out with descriptors for chickpea (*Cicer arietinum* L.)^60^ according to methods in routine use at VIR^11^ and ICARDA^61^.

### RAD sequencing and SNP-calling

Genomic DNA was digested with two restriction enzymes, *Hind*III and *Nla*III. Two different types of adapters were used in this protocol. The “barcode” adapter was ligated to the end generated by *HindIII* allowing pooling the samples. The second adapter called “common” adapter was ligated to the overhang end of *NlaIII*. We performed a selection size and 14 rounds of PCR was used to amplify the fragments. Fragments were sequenced as 100 base reads on an Illumina HiSeq4000 at the University of California at Davis Genome Core. All Illumina data is available in NCBI under the BioProjects PRJNA353637 and PRJNA388691. Illumina reads were mapped to the *Cicer arietinum* CDCFrontier reference^62^ using BWA MEM^63^ under default mapping parameters. Polymorphisms were called using the GATK pipeline^64^, which considers indel realignment and base quality score recalibration, and calls variants across all samples simultaneously through the HaplotypeCaller program in GATK. Variants were filtered using standard hard filtering parameters according to GATK Best Practices recommendation^65, 66^. More precisely, GBS data was filtered to only retain SNP calls with Mapping Quality (MQ) > 37 and Quality by Depth (QD) > 24. Both metrics take into consideration the quality of the mapping and genotype calls to ensure that only those with highest confidence were used. The SNPs were also filtered to retain those with MQRankSum < |2.0|, which ensure that there is no difference in the Mapping Quality scores for both alleles. This filtering removed nearly 60% of variant sites reported by GATK and only retained those that pass all three criteria. For this SNP calls we then used VCFtools^67^ to implement the following inclusion criteria: minor allele frequency (MAF) more than 3%, genotype call-rate more than 90%, and Hardy-Weinberg Equilibrium (HWE) exact P-value more than 10^−5^. Overall, 14059 SNPs remain to further analysis.

### Phenotype data analysis

Factor analysis (“varimax” method) was performed using the “psych” R package (R version 3.3.1 was used). The significance of correlations between factors, geographic distribution and bioclimatic variables was tested using the “corrgram” R package.

### Genotype data analysis

Principal component analysis was conducted using the “SNPRelate” R package. VCFtools^67^ was used to calculate the squared correlation coefficient between genotypes to construct LD plots, with LD decay computed according to Hill and Weir^68^. With this tool we also constructed Depth plot (see Supplementary Figure S5) and four landraces were excluded due to low coverage. Relationships among accessions were calculated and the maximum likelihood phylogenetic trees were constructed using SNPhylo^69^. To assess number of clusters (K) in population structure we run STRUCTURE^70^ program with 10 replicates for each K from 1 to 10, using 100,000 burnin period and 100,000 MCMC repeats after burnin. Then Evanno’s test^71^ implemented in Harvester^72^ was used to evaluate the best number of clusters. The results of Evanno’s test showed that the best estimation of K parameter is 2. Although separation in these clusters is reflected in separation of first principal component on the principal component plot, we haven’t found any biological meaning of the clusters. So we choose K = 3 which is suboptimal, but the separation coincides with well-resolved clusters of Fig. 1 seen on both PCA plot and phylogenetic trees. In order to do GWAS analysis and heritability estimation, genetic data was converted into FastLMM^73^ and GCTA^74^ formats using the PLINK toolset^75^. GWAS analyses were performed using the FASTLMM toolset (Factored Spectrally Transformed Linear Mixed Models). Q-values (which are adjusted p-values calculated using an optimised FDR approach^76^) were calculated and a q-value threshold of <0.05 (corresponding to p-value < 3.761e-05) was used to determine significant SNPs. In order to assess LD blocks (confidence interval for LD [0.7, 0.99]), covering significant SNPs, we used the Haploview^77^. The GCTA program was used to estimate explained variance of polymorphisms (see Supplementary Table S2).

### Bioclimatic Analysis

Environmental data were downloaded in the form of GIS layers from the WorldClim – Global Climate Data^78^ and USGS^79^. Layers describe a combination of current conditions and interpolations of observed values that span 1950–2005. Digital Elevation Data, GTOPO30, were downloaded from the NASA – USGS LP DAAC archive (Global 30 Arc-Second Elevation)^80^. The ‘land suitability for cultivation’ dataset was downloaded from the Nelson Institute, Centre for Sustainability and the Global Environment, University of Wisconsin-Madison, (SAGE)^81, 82^. Koppen-Geiger climate zones were acquired from the Center for International Development at Harvard University^83^. Data layers that came in vector format were rasterized to match a spatial resolution of 30 sec, which corresponds to approximately 1 sq km at the equator. The data were interpolated from average monthly recordings from weather stations^84^. The data layers selected for the current study are given in Supplementary Table S5. Both environmental layers and accession points were in the Longitude/Latitude coordinate system with WGS84 datum. The environmental values for each accession point were extracted from corresponding layers using the extraction tool in ESRI ArcGIS software^73, 85^.

## Electronic supplementary material

Supplementary information

Supplementary Table S3

Supplementary Table S4

Phenotypes+enviroment
