# Supplementary material for: Genomic and phenotypic analysis of Vavilov’s historic landraces reveals the impact of environment and genomic islands of agronomic traits
Source: Sci Rep. 2017 Jul 6;7:4816. doi: 10.1038/s41598-017-05087-5 (PMC5500531; doi:10.1038/s41598-017-05087-5)
Supplement: Supplementary file 1 — Supplementary information [file 41598_2017_5087_MOESM1_ESM.pdf]

Supplementary information

Genomic and phenotypic analysis of Vavilov's historic landraces reveals the impact of environment and genomic islands of agronomic traits

Plekhanova, Elena[1], Margarita A. Vishnyakova[2], Sergey Bulyntsev[2], Peter L. Chang[3,4], Noelia Carrasquilla-Garcia[3], Kassaye Negash[3], Eric von Wettberg[5], Nina Noujdina[6], Douglas R. Cook[3], Maria G. Samsonova[1], and Sergey V. Nuzhdin[4,1*]

[1]Department of Applied Mathematics, St. Petersburg State Polytechnical University, St. Petersburg, Russia
[2]Federal Research Centre All-Russian N.I. Vavilov Institute of Plant Genetic Resources (VIR), St. Petersburg, Russia
[3]Department of Plant Pathology, University of California at Davis, Davis, CA
[4]Program Molecular and Computation Biology, Dornsife College of Letters, Arts, and Sciences, University of Southern California, Los Angeles, CA
[5]Department of Biological Sciences and International Center for Tropical Botany, Florida International University, Miami, FL
[6]School of Architecture, University of Southern California, Los Angeles, CA.

*Communicating Author
RRI Hall, Molecular and Computation Biology
1050 Childs Way
University of Southern California
Los Angeles, CA 90089
Phone: (213) 740 5773
Fax: (213) 740 8660
e-mail: snuzhdin@usc.edu

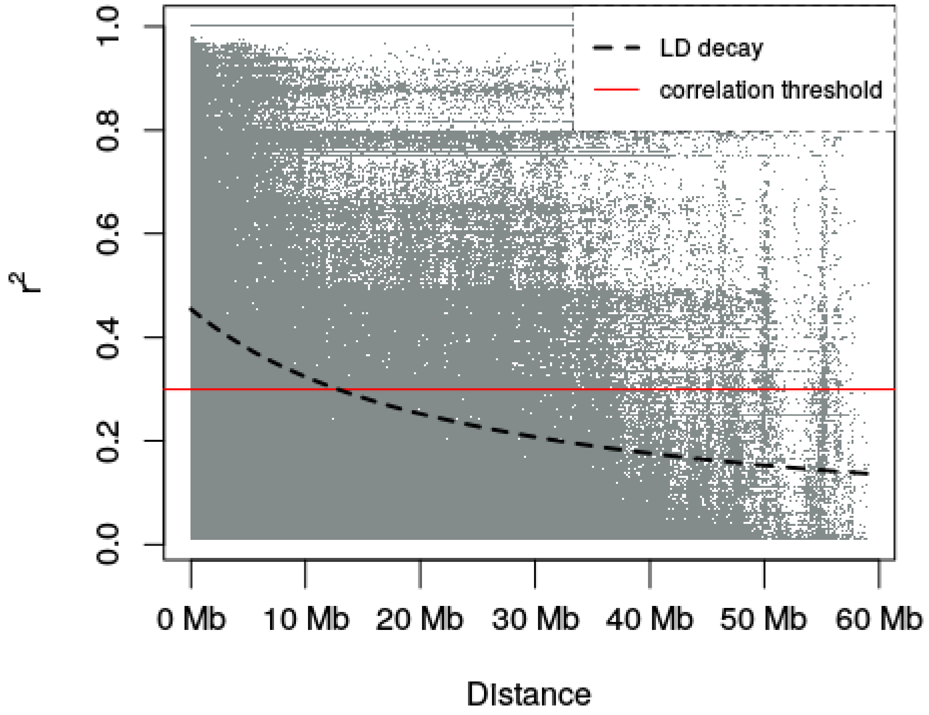

Supplementary Figure S1. Decrease in LD measured by correlation-squared (r^2) as a function of genetic distance between SNPs for landraces from Ethiopia.

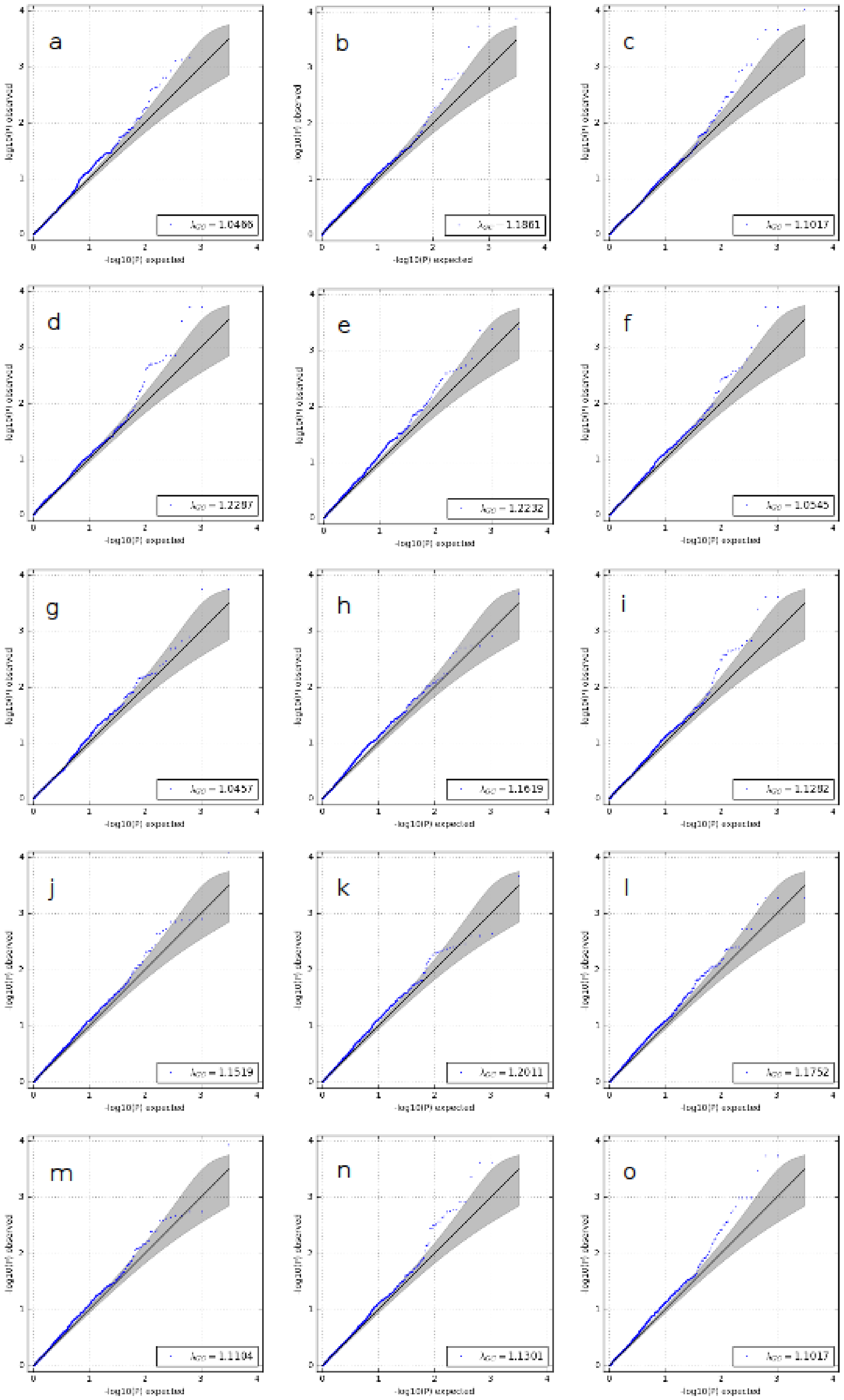
a
λ_{GC} = 1.0466
b
λ_{GC} = 1.1861
c
λ_{GC} = 1.1017
d
λ_{GC} = 1.2287
e
λ_{GC} = 1.2232
f
λ_{GC} = 1.0545
g
λ_{GC} = 1.0457
h
λ_{GC} = 1.1619
i
λ_{GC} = 1.1282
j
λ_{GC} = 1.1519
k
λ_{GC} = 1.2011
l
λ_{GC} = 1.1752
m
λ_{GC} = 1.1104
n
λ_{GC} = 1.1301
o
λ_{GC} = 1.1017
-log10(P) expected
-log10(P) observed

Supplementary Figure S2. SNP QQ-plots for Syrian phenotype data. The phenotypes are: angle of primary branches (a), days of flowing (b), days from sowing to start flowering (c), days from sowing till maturity (d), plant dry weight without seeds (e), plant with seeds and roots dry weight (f), flower colour (g), height of lower pod attachment (h), Stem height (i), number of pods per plant (j), number of seeds per plant (k), weight of 1000 seeds (l), seeds weight per plant (m), seeds weigth to plant dry weight ratio (n), plant canopy width (o).

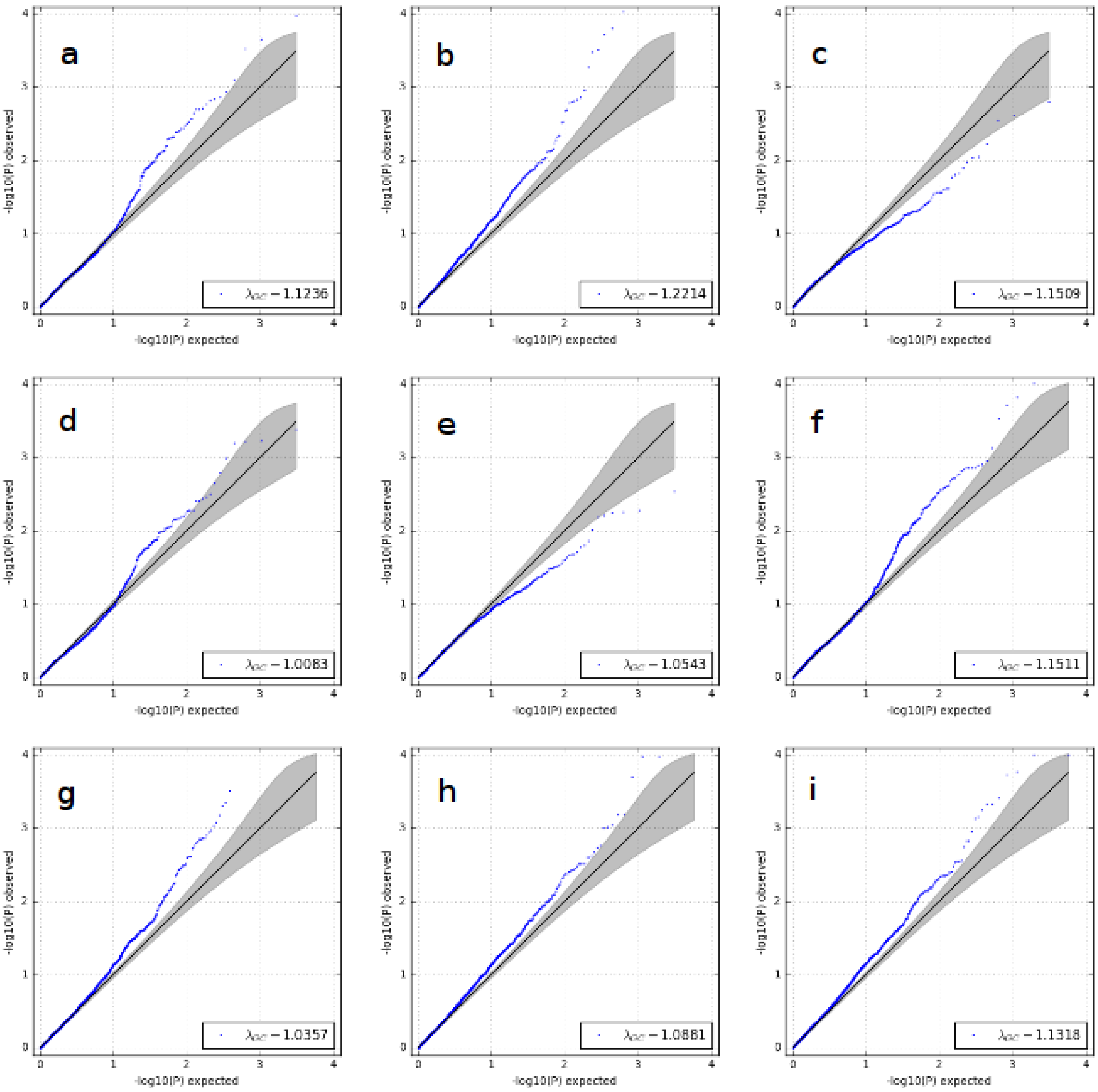

Supplementary Figure S3. SNP QQ-plots for Astrakhan phenotype data. The phenotypes are: Ascochyta resistance (a), bush shape (b), lower pod height (c), number of seeds per plant (d), seed color (e), seed shape (f), seed size (g), days from sowing to start flowering (h), days from sowing till maturity (i).

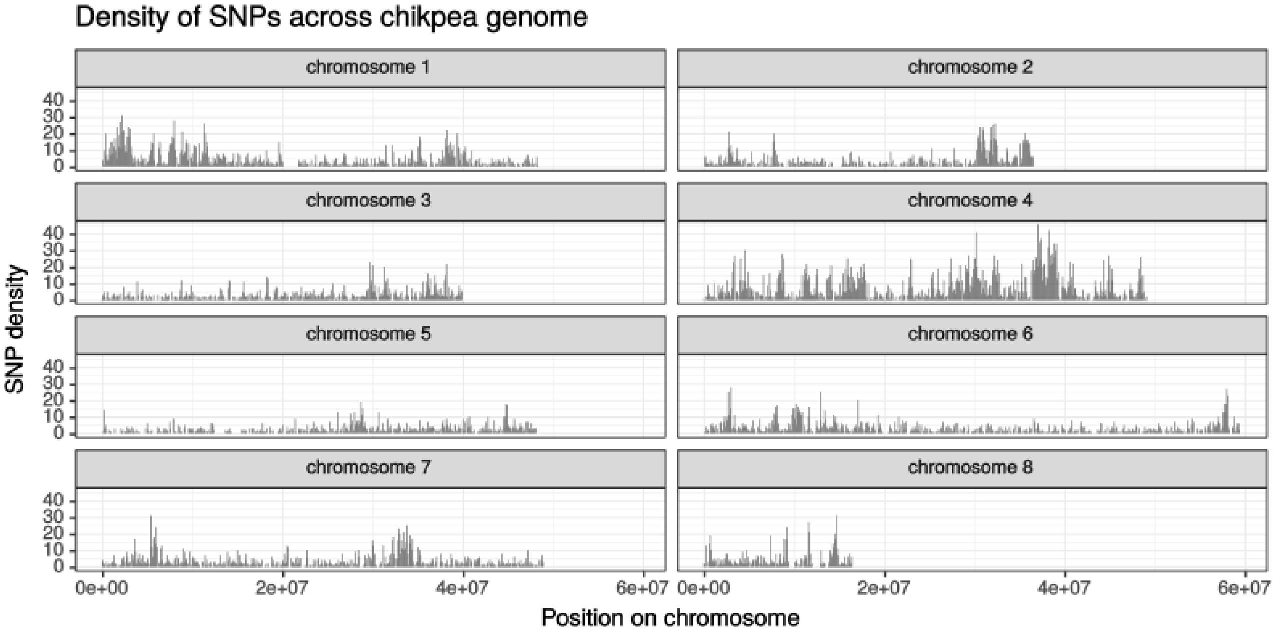

Supplementary Figure S4. Density of SNPs across the chickpea genome.

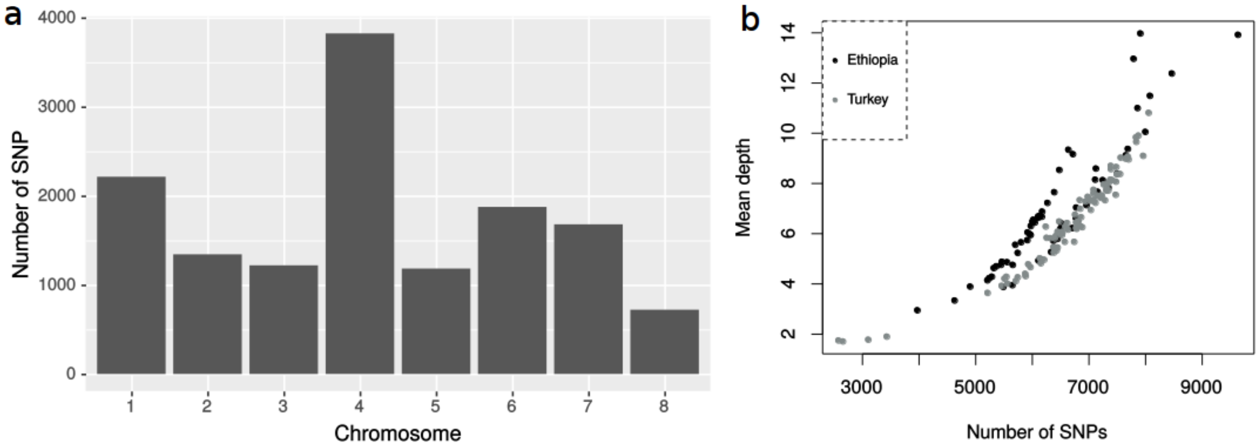

Supplementary Figure S5. Analysis of genotypic data. (a) Distribution of SNPs along 8 chromosomes of chickpea genome. (b) Mean depth (number of SNPs) per accession. Accessions denoted by dots fall in two clusters corresponding to country of origin, Turkey or Ethiopia.

Supplementary Table S1. Number of accessions from two countries of origin (Ethiopia and Turkey), for which we have phenotype data (collected in Astrakhan and Syria). All accessions phenotyped in Syria were phenotyped in Astrakhan too.

	Phenotyped in Syria	Phenotyped in Astrakhan	Total*
Ethiopia	21	57	63
Turkey	48	52	84
Total	69	109	147

* Total number of accessions from the country of origin. We don't have phenotype data for part of them.

Supplementary Table S2. Proportion of variance of Syrian and Astrakhan phenotype data and bioclimatic variables, explained by genotype.

Type	Phenotype	Genotype variation (SE)	Phenotype variation (SE)	beta* (SE)	p-value
Syria	Number pods per plant	0.177 (0.110)	0.814 (0.145)	0.218 (0.139)	0.001486
	Number seeds per plant	0.026 (0.017)	0.148 (0.026)	0.178 (0.121)	0.002134
	Plant dry weight without seeds	0.345 (0.172)	0.993 (0.176)	0.343 (0.169)	1.049e-05
	Seed weight per plant	0.054 (0.038)	0.348 (0.062)	0.156 (0.113)	0.004758
	Plant with seeds and roots dry weight	0.061 (0.031)	0.178 (0.031)	0.344 (0.177)	0.00003
	Weight of 1000 seeds	0.123 (0.033)	0.209 (0.036)	0.587 (0.118)	1.11e-16
	Days from sowing till maturity	0.201 (0.059)	0.326 (0.057)	0.617 (0.136)	2.247e-08
	Plant canopy width	0.238 (0.133)	0.662 (0.116)	0.359 (0.201)	0.001418
	Height of lower pod attachment	0.099 (0.030)	0.159 (0.028)	0.625 (0.143)	1.544e-10
	Stem height	0.164 (0.074)	0.357 (0.062)	0.459 (0.195)	7.457e-06
	Flower colour	0.304 (0.088)	0.581 (0.101)	0.523 (0.120)	1.295e-09
	Factor 1	0.218 (0.052)	0.291 (0.052)	0.749 (0.096)	7.829e-13
Astrakhan	Seed size	0.071 (0.026)	0.159 (0.022)	0.441 (0.146)	6.994e-15
	Seedling-flowering	0.131 (0.097)	0.823 (0.137)	0.159 (0.120)	0.002655
	Ascohyta tolerance	0.038 (0.034)	0.416 (0.068)	0.092 (0.084)	0.0197
Bioclimatic variables	Elevation	0.169 (0.055)	0.349 (0.048)	0.485 (0.133)	3.463e-06
	Annual Mean Temp	0.168 (0.083)	0.849 (0.117)	0.198 (0.096)	2.361e-08
	Temp Mean Diurnal Range	0.062 (0.028)	0.197 (0.027)	0.315 (0.136)	9.408e-08
	Temp Annual Range	0.109 (0.023)	0.152 (0.219)	0.715 (0.087)	3.047e-10
	Annual Precipitation	0.189 (0.075)	0.531 (0.073)	0.358 (0.131)	9.823e-13
	Precipitation Seasonality	0.273 (0.109)	0.558 (0.789)	0.489 (0.168)	1.831e-11

* beta - ratio of genetic variance to phenotypic variance of a trait.

Supplementary Table S3. Significant SNPs for Syrian, Astrakhan phenotypes and for Syrian phenotypes including two first components as a cofactor.

(see Supp_Table3.xls)

Supplementary Table S4. Putative functions of the genes with significant SNPs. The information about most similar protein sequence, InterPro domains, GO ontology terms is taken from the Legume IP database[1].

(see Supp_Table4.xls, corresponding references are in Supplementary Reference section)

Supplementary Table S5. The correlations between bioclimatic variables and factors of Astrakhan phenotype data.

Bioclimatic variable	FA1	FA2	FA3
Mean Diurnal Temperature Range	-0.55**	-0.11	0.04
Temperature Seasonality	0.69**	0.10	-0.11
Temperature Annual Range	0.62**	0.06	-0.12
Annual Precipitation	-0.46**	-0.11	-0.07
Precipitation of Driest Quarter	-0.46**	-0.19	0.09

** - significance p-value < 0.01; correlations with Annual Mean Temperature, Elevation, Kopper-Geiger climate zones, Land Suitability for Cultivation were insignificant for all the factors.

References

1. Li, J., Dai, X., Liu, T. & Zhao, P. X. LegumeIP: An integrative database for comparative genomics and transcriptomics of model legumes. *Nucleic Acids Res.* **40,** 1221–1229 (2012).
2. Lin, F.-Y., Lu, Q.-X., Xu, J.-H. & Shi, J.-R. Cloning and expression analysis of two salt and Fusarium graminearum stress associated UDP-glucosyltransferases genes in wheat. *Yi Chuan* **30,** 1608–1614 (2008).
3. Ogawa, T. *et al.* Modulation of NADH Levels by Arabidopsis Nudix Hydrolases, AtNUDX6 and 7, and the Respective Proteins Themselves Play Distinct Roles in the Regulation of Various Cellular Responses Involved in Biotic/Abiotic Stresses. *Plant Cell Physiol.* **57,** 1295–1308 (2016).
4. Umate, P., Tuteja, R. & Tuteja, N. Genome-wide analysis of helicase gene family from rice and Arabidopsis: A comparison with yeast and human. *Plant Mol. Biol.* **73,** 449–465 (2010).
5. Klaus-Heisen, D. *et al.* Structure-function similarities between a plant receptor-like kinase and the human interleukin-1 receptor-associated kinase-4. *J. Biol. Chem.* **286,** 11202–11210 (2011).
6. Alvarez, M. E., Nota, F. & Cambiagno, D. A. Epigenetic control of plant immunity. *Molecular Plant Pathology* **11,** 563–576 (2010).
7. Moreau, M., Tian, M. & Klessig, D. F. Salicylic acid binds NPR3 and NPR4 to regulate NPR1-dependent defense responses. *Cell Res.* **22,** 1631–1633 (2012).
8. Delorge, I., Janiak, M., Carpentier, S. & Van Dijck, P. Fine tuning of trehalose biosynthesis and hydrolysis as novel tools for the generation of abiotic stress tolerant plants. *Front. Plant Sci.* **5,** 147 (2014).
9. Marcos, R. *et al.* 9-Lipoxygenase-derived oxylipins activate brassinosteroid signaling to promote cell wall-based defense and limit pathogen infection. *Plant Physiol.* **4,** 2324–2334 (2015).
10. Singh, A., Breja, P., Khurana, J. P. & Khurana, P. Wheat Brassinosteroid-Insensitive1 (TaBRI1) interacts with members of TaSERK gene family and cause early flowering and seed yield enhancement in arabidopsis. *PLoS One* **11,** e0153273 (2016).
